# Changes in colloid oncotic pressure during cardiac surgery with different prime fluid strategies

**DOI:** 10.1177/02676591231193626

**Published:** 2023-08-08

**Authors:** Anne Maria Beukers, Juan de Villiers Hugo, Renard Gerardus Haumann, Jan Willem Taco Boltje, Evy Loan Khiam Ie, Stephan Alexander Loer, Carolien Suzanna Enna Bulte, Alexander Vonk

**Affiliations:** 1 Department of Anaesthesiology, 1209 Amsterdam UMC location Vrije Universiteit Amsterdam, Amsterdam, The Netherlands; 2 Amsterdam Cardiovascular Sciences, Amsterdam, The Netherlands; 3 Department of Cardiothoracic Surgery, LUMC, 4501 Leiden University, Leiden, The Netherlands; 4 Department of Cardiothoracic Surgery, Thoracic Center Twente, Enschede, The Netherlands; 5 Department of Cardiothoracic Surgery, 26066 Amsterdam UMC location University of Amsterdam, Amsterdam, The Netherlands

**Keywords:** colloid oncotic pressure, cardiopulmonary bypass, haemodilution, prime fluid, fluid extravasation

## Abstract

**Objective:**

In cardiac surgery, colloid oncotic pressure (COP) is affected by haemodilution that results from composition and volume of prime fluid of cardiopulmonary bypass (CPB). However, the extent to which different priming strategies alter COP is largely unknown. Therefore, we investigated the effect of different priming strategies on COP in on-pump cardiac surgery.

**Methods:**

Patients (*n* = 60) were divided into 3 groups (*n* = 20 each), based on the center in which they were operated and the specific prime fluid strategy used in that center during the inclusion period. CPB prime fluids were either gelofusine-, albumin-, or crystalloid based, the latter two with or without retrograde autologous priming.

**Results:**

In all groups, COP was lowest after weaning from CPB and one hour after CPB. Between groups, COP was lowest with gelofusine prime fluid (16.4, 16.8 mmHg, respectively) compared with crystalloids (MD: -1.9; 95% CI:-3.6, -0.2; *p* = .02 and MD: -2.4, 95% CI: -4.2, -0.7; *p* = .002) and albumin (MD: -1.8, 95% CI: -3.5, -0.50; *p* = .041 and MD: -2.4, 95% CI: -4.1, -0.7; *p* = .002). In all groups, the decrease in COP one hour after bypass compared to baseline correlated positively with fluid balance at the end of surgery (*p* < .001).

**Conclusions:**

COP significantly decrease during CPB surgery with the largest decrease in COP at the end of surgery, while at the same time fluid balance increases. We suggest that prime fluid strategy should be carefully selected when maintenance of COP during cardiac surgery is desirable.

## Introduction

Colloid oncotic pressure (COP), determined by all plasma proteins in intra- and extravascular compartments, plays a key role in transcapillary fluid movement and interstitial fluid accumulation.^1^ During cardiac surgery, priming of the cardiopulmonary bypass (CPB) results in significant haemodilution, thereby affecting COP and possibly compromising tissue oxygenation and organ perfusion.^2^

Traditionally, CBP priming fluid consists of crystalloids, colloids or a combination of both. Crystalloids such as Ringers and lactated Ringers increase interstitial fluid accumulation due to an osmotic effect.^3^ Colloids (i.e. gelofusine) increase COP more than crystalloids, resulting in lower fluid requirements in cardiac surgery with CPB.^4^ On the other hand, artificial colloids (hydroxyethyl starch specifically) may have negative effects on haemostasis, i.e. decreased platelet function,^5^ increased blood loss and transfusion requirements.^6^ Besides crystalloids or colloids, human albumin can be added to the prime which protects the endothelial glycocalyx^7^ and has beneficial effects on platelet count in cardiac surgery.^4^ Another frequently used addition is retrograde autologous priming (RAP), which reduces the effect of haemodilution as well as compensates for the drop in COP during on-pump cardiac surgery. The use of RAP allows a reduction in transfusion rates^8,9^ and is included in the guidelines on patient blood management for adult cardiac surgery^10^ and recommended in the guidelines on CPB in adult cardiac surgery.^11^

The optimal composition of prime fluid is unknown, in particular the type and amount of crystalloid or colloid fluids used.^11^ Data from our unpublished survey (2019) among all cardiac surgery centers in the Netherlands (response 13 out of 16) showed a heterogeneous priming strategy, which differed in all centers. Fluids chosen for CPB were gelofusine, albumin, HES and crystalloids either with RAP. Investigating changes in COP could help determine the choice for CPB priming with respect to minimal interstitial fluid accumulation and preserved organ perfusion and function. As a first step, this study investigated the changes in COP in patients undergoing elective cardiac surgery with *currently* used CPB prime fluids: gelofusine, albumin or crystalloids, the last two either combined with RAP.

## Methods

This prospective observational study was pre-registered in the Netherlands Trial Register (NL9055) and approved by the Human Subjects Committee of the Amsterdam University Medical Center (Amsterdam, the Netherlands, approval number 20-463). This study conformed to the reporting ICMJE Recommendations for the Protection of Research participants. This study was performed in two University Medical Centers in the Netherlands, Amsterdam UMC location VU medical center Amsterdam and LUMC, precisely due to their differences in priming strategies. Written informed consent was obtained from all subjects before inclusion. Adult patients for coronary artery bypass graft surgery with CPB, valve surgery or a combination of both were included. Exclusion criteria were emergency surgery, previous heart surgery, and elective thoracic aortic surgery.

### Study groups

Patients were divided into 3 different groups, based on the center in which they were operated and the specific prime fluid strategy used in that center during the inclusion period. As this was an observational trial, patients were not randomized in groups.

Group 1 (gelofusine, *n* = 20): 750 mL modified fluid gelatin (Braun Melsungen AG, Germany), 500 mL lactated Ringers (Baxter BV, Utrecht, the Netherlands), 50 mL sodium bicarbonate (8.4%, Braun Melsungen, AG, Germany), and 100 mL mannitol (15%, Baxter BV, Utrecht, the Netherlands) (gelofusine group), Group 2 (albumin, *n* = 20): 100 mL human albumin (20%, Sanquin, Amsterdam, the Netherlands), 900 mL Ringers, 5000 IE heparin, and 100 mL mannitol (15%) (albumin group), Group 3 (crystalloids, *n* = 20): 1000-1200 mL Ringers, 5000 IE heparin and 100 mL mannitol (15%) (crystalloid group). RAP was performed in the albumin and crystalloid groups at the discretion of the attending perfusionist. The anesthesia and CPB protocols of both institutions are described in Appendixes 1 and 2.

### Colloid oncotic pressure

Colloid oncotic pressure was measured using an oncotic cell (Osmomat 050, Gonotec, Berlin, Germany). Arterial blood samples were collected and centrifuged to obtain plasma. These plasma samples were stored at 4°C before measurements within 7 days. Detailed description of COP measurements is described in Appendix 3. COP was also calculated (Landis and Pappenheimer formula).^12^ Blood samples were taken at six time points: after induction of anesthesia (T1), aortic cross clamping (T2), weaning from CPB (T3), 1 h after weaning from CPB (T4), arrival at intensive care unit (ICU) (T5), and 6 h after admission to the ICU (T6).

### Secondary parameters

Secondary parameters included albumin, total protein, haematocrit, platelet count, transfusion requirements, fluid balance and –requirements and postoperative weight gain.

Patient characteristics included age, gender, body mass index, body surface area, smoking, diabetes on medication, comorbidities, European system for cardiac operative risk evaluation (EuroSCORE), type of surgery, CPB- and aortic cross clamping duration, blood loss, lactate levels, urea levels, estimated glomerular filtration rate, creatinine clearance, use of vasoactive medication, postoperative haemostatic, renal and volume related parameters as included intraoperative, duration of mechanical ventilation and complications.

### Statistics

Sample size calculation was based on data from a pilot study (unpublished) in which a standard deviation (SD) for COP of 2.3 mmHg was found. A mean decrease in COP of 1 mmHg (1h after weaning from CPB compared with the preoperative period) correlated with a mean increase in fluid balance (275 mL, 95% CI: 22-529 mL; *p* = .035). To detect a difference in fluid balance of 550 mL between three study groups based on 6 pairwise comparisons between four time points, the two-sided significance level was set at 0.05/0.00,833 (Bonferroni correction). Consequently, a sample size of 20 patients per group (including 20% dropout) was required. As no other additional secondary parameters were measured in our pilot study, new study data were required.

Data were stored in an electronic case report form in Castor (EDC, 2020) and analysed using SPSS (26.0; IBM, New York, USA). Data were locked before analysis. Data are presented as percentage (%), mean ± SD or median ± interquartile range (IQR) for non-normally distributed variables. Normality was checked using normal probability plots. Means between groups were compared with a one-way ANOVA, medians with a non-parametric independent-samples median test and frequencies with a Chi-square test. Mean COP levels were compared using linear mixed models. The models included time as fixed factor and patient as random effect. With significant time factor, means for different time points were compared pairwise using posthoc tests with Bonferroni-adjusted significance levels. Similar analyses were performed for the other repeatedly measured continuous variables. For non-normally distributed outcome variables, log-transformations were performed before mixed linear models. Linear regression was used for correlations between two continuous variables. A *p*-value <.05 was considered statistically significant. Finally, intraclass correlation (two-way mixed for single measures with absolute agreement) was used to compare measured and calculated COP (<0.5 indicated pore reliability; 0.5-0.75,moderate reliability; 0.75-0.9, good reliability; >0.9, excellent reliability).^13^

## Results

Patients were included from December 2020 to July 2021. COP measurements were complete, except for 15 measurements at time points 5 and/or 6 due to evening restrictions during the COVID pandemic. Two patients were transferred to another hospital due to lack of intensive care beds.

Table 1 shows patient and surgical characteristics. Patients did not differ in terms of comorbidities, except for smoking and other relevant comorbidities (Table 1). No differences were observed with regard to antiplatelet therapy (Supplements, Table 1). Table 2 shows intraoperative results. Groups differed in terms of prime volume and cardioplegia type (Table 2, *p* < .001, .001 respectively).

**Table 1.** Baseline characteristics.

	Gelofusine (*n* = 20)	Crystalloids (*n* = 20)	Albumin (*n* = 20)	*p*-value
Patient characteristics				
Gender, male	17 (85%)	14 (70%)	12 (60%)	0.210
Age, years	66.9 ± 11.6	65.7 ± 8.3	66.6 ± 8.6	0.921
Body Mass index, kg.m‾^2^	26.9 ± 4.0	28.9 ± 4.7	26.4 ± 3.5	0.139
Body surface area, m^2^	2.01 ± 0.22	2.01 ± 0.23	1.92 ± 0.24	0.318
Comorbidities				
None	3 (15%)	3 (15%)	6 (30%)	0.392
Smoker	2 (10%)	8 (40%)	2 (10%)	0.024*
Hypertension	11 (55%)	12 (60%)	9 (45%)	0.626
Chronic obstructive pulmonary disease	1 (5%)	1 (5%)	0	0.596
Hypercholesterolemia	10 (50%)	7 (35%)	7 (35%)	0.535
Diabetes type I	0	0	0	—
Diabetes type II	1 (5%)	5 (25%)	6 (30%)	0.112
Other#	12 (60%)	4 (20%)	3 (15%)	0.004*
Surgical characteristics				
CABG	12 (60%)	13 (65%)	11 (55%)	0.812
Mitral valve repair/replacement	1 (5%)	1 (5%)	1 (5%)	1.000
Aortic valve replacement	5 (25%)	5 (25%)	7 (35%)	0.720
CABG+Valve	2 (10%)	1 (5%)	2 (10%)	0.804
CPB Time, min	112.7 ± 35.4	114.8 ± 33.0	129.0 ± 40.7	0.314
AoX time, min	79.0 ± 27.7	84.8 ± 28.6	95.5 ± 28.0	0.180
Total surgery time, min	262.0 ± 70.7	234.1 ± 52.6	264.5 ± 55.6	0.217
Blood cardioplegia	0	19 (95%)	19 (95%)	<0.001*
EuroSCORE	4.72 ± 3.61	3.93 ± 3.01	3.68 ± 2.02	0.512
ASA-score	3.05 ± 0.22	2.75 ± 0.44	2.85 ±0.49	0.065

#Other: Coronary artery disease, non-significant valve disease, history of myocardial infarction, atrial fibrillation, extra-coronary arteriopathy, nephropathy, obstructive sleep apnea syndrome, auto-immune diseases.Means compared with one-way ANOVA. Frequencies compared with Chi-square test *statistically significant value *p* < .05.

**Table 2.** Results.

	Gelofusine (*n* = 20)	Crystalloids (*n* = 20)	Albumin (*n* = 20)	*p*-value
Intraoperative				
Total prime volume, ml	1323 ± 80	1015 ± 75	1200 ± 97	<0.001*
†RAP, ml	0 [0-0]	216 ± 217	224 ± 183	<0.001*
†Prime minus RAP, ml	1350 [1350-1350]	799 ± 239	976 ± 239	<0.001*
†Prime: Crystalloids, ml	484 ± 35	1000 [1000-1000]	980 ± 89	0.014*
†Prime: Gelofusine, ml	729 ± 45	—	—	<0.001*
†Prime: Human albumin, ml	0 [0-0]	—	100 [100-100]	<0.001*
†Crystalloid cardioplegia, ml	1725 ± 453	0 [0-0]	0 [0-0]	<0.001*
Additional fluids, ml	1681 ± 449	964 ± 487	1157 ± 446	0.019*
Additional crystalloids, ml	1290 ± 556	959 ± 493	1099 ± 438	0.118
†Additional colloids, ml	148 ± 145	0 [0-0]	58 ± 71	0.001*
Fluid balance, ml	1452 ± 1062	598 ± 707	780 ± 538	0.003*
†PRBCs, ml	0 [0-140]	0 [0-0]	0 [0-206]	0.727
Platelet transfusion, ml	83 ± 153	95 ± 148	106 ± 148	0.648
†Fresh frozen plasma, ml	—	0 [0-0]	0 [0-0]	0.501
Cell saver volume, ml	655 ± 238	111 ± 182	294 ± 232	<0.001*
Total vasoactive medication end of surgery				
†Noradrenaline, mcg	465 [188-968]	26 [0-124]	19 [0-112]	0.001*
†Dopamine, mg	0 [0-10]	—	—	0.001*
†Dobutamin, mg	—	0 [0-0]	0 [0-0]	0.059
†nitroglycerin,mcg	—	0 [0-25]	0 [0-44]	0.008*
†Phenylephrine, mcg	225 [100-500]	670 ± 486	724 ± 398	0.016*
†Ephedrine, mg	0 [0-0]	11 ± 10	8 ± 12	<0.001*
Postoperative				
Additional fluids, ml	937 ± 710	829 ± 431	1161 ± 697	0.240
†PRBCs, ml	—	—	0 [0-0]	0.106
†Platelet transfusion, ml	0 [0-160]	0 [0-0]	195 ± 306	0.166
†Fresh frozen plasma, ml	—	0 [0-0]	204 ± 333	0.042*
Duration of mechanical ventilation, h	6.7 ± 4.1	1.8 ± 1.8	4.0 ± 3.9	<0.001*

Means compared with one-way ANOVA. †Medians compared with non-parametric independent-samples median test. Frequencies compared with Chi-square test *statistically significant value *p* < .05.

### Colloid oncotic pressure

**Figure 1.**
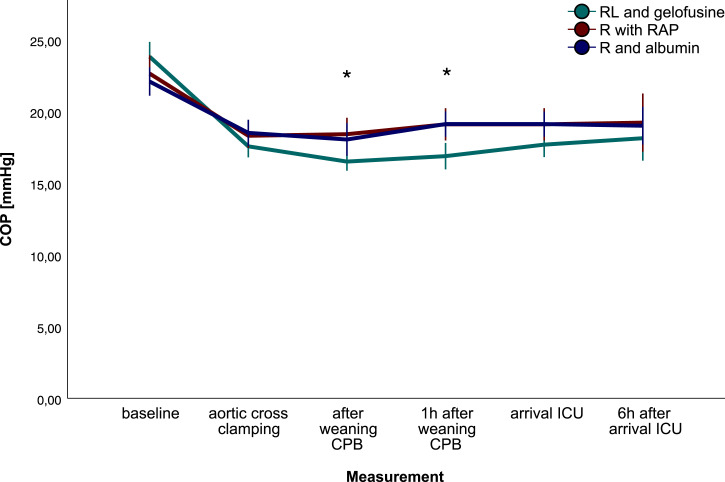
Colloid oncotic pressure.

COP, displayed in Figure 1, was comparable after induction of anesthesia (23.7, 22.6, and 22.2 mmHg; gelo vs. cryst vs. alb respectively), but decreased after initiation of CPB in all groups. COP differed between groups over time (*p* < .001), also after correction for BSA (*p* = .024). COP was lowest after weaning from CPB and one hour after CPB with gelofusine prime fluid (16.4, 16.8 mmHg, respectively) compared with crystalloids (mean difference [MD]: -1.9; 95% confidence interval [CI]:-3.6, -0.2; *p* = .020 and MD: -2.4, 95% CI: -4.2, -0.7; *p* = .002) and albumin (MD: -1.8, 95% CI: -3.5, -0.5; *p* = .041 and MD: -2.4, 95% CI: -4.1, -0.7; *p* = .002). Postoperative COP was comparable between groups. Moderate reliability was found between calculated and measured COP (intraclass correlation 0.650).

### Fluid accumulation

Fluid balance at the end of surgery was higher with gelofusine, compared with crystalloids and albumin prime fluid (Table 2, *p* = .003). Additional crystalloid fluids were comparable between groups (*p* = .118), while additional colloid fluids were higher with gelofusine and albumin prime fluid compared with crystalloids (*p* = .001, Table 2). A negative correlation was observed between delta (∆) COP (one hour after weaning vs. baseline) and fluid balance (r^2^ = 0.453, *p* < .001), fluid requirements (*p* < .001) and duration of mechanical ventilation (*p* = .002).

### Haemodilution and blood product use

**Figure 2.**
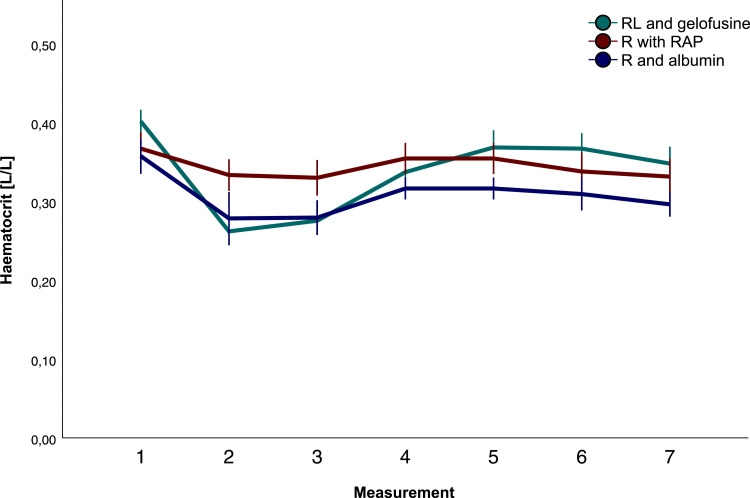
Haematocrit.

Haematocrit differed between groups during surgery and during ICU stay (*p* < .001, Figure 2). After cross clamping the aorta, haematocrit was preserved with crystalloids compared with colloid groups (gelo *p* < .001 and alb *p* < .001) and remained higher until weaning from CPB (cryst vs. gelo, *p* < .001; cryst vs. alb, *p* = .001). On arrival at the ICU haematocrit was highest with gelofusine and crystalloids than with albumin (gelo vs. alb *p* = .01, and cryst vs. alb *p* = .020). Until the next morning, haematocrit remained highest with gelofusine compared with albumin (Figure 2, T6 *p* < .001 and T7 *p* = .001). Transfused volume of cell saver blood was highest with gelofusine (655 ± 238 vs. 111 ± 182 vs. 294 ± 232 mL, gelo vs. cryst vs. alb respectively, *p* < .001). Haemoconcentration was not performed in any of the groups. Packed red blood cell (PRBC) requirements were comparable between groups intraoperatively (*p* = .727) and postoperatively (*p* = .106). Postoperatively, fresh frozen plasma administration was highest in the albumin group (Table 2, *p* = .042).

### Other secondary outcome variables

**Figure 3.**
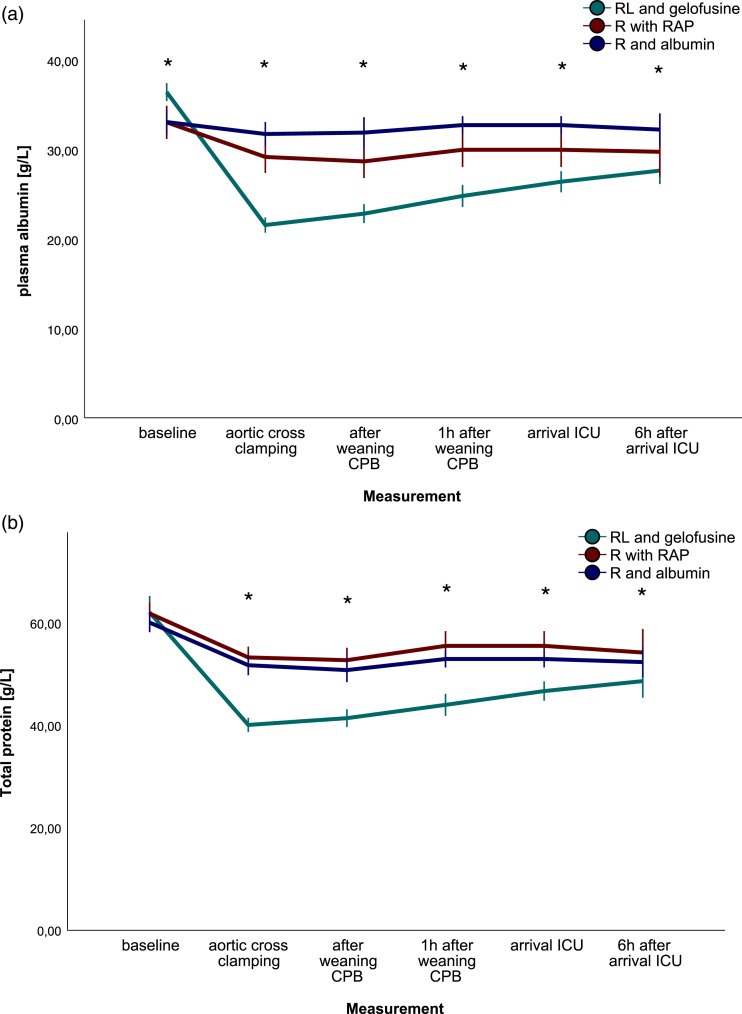
Plasma albumin and total protein.

Plasma albumin (Figure 3, *P* < .001) and total protein (Figure 3, *P* < .001) differed between groups over time. No differences were observed in blood loss, platelet count, lactate levels, and creatinine (*p* = .181, .244, 0.171, 0.730 respectively).

### Adverse events

Two patients in the gelofusine group had a cerebral vascular accident, one most likely due to pre-existing atrial fibrillation. The second due to pre-existing carotid stenosis and was successfully treated with a carotid endarterectomy. Unfortunately, one patient died due to respiratory insufficiency after a postoperative cardiac arrest. One patient tested positive for COVID-19 postoperatively without consequences for ICU or hospital admission. Until hospital discharge non-pre-existing atrial fibrillation, reoperation for bleeding and length of hospital stay were comparable between groups (*p* = .330, .574, 0.557 respectively). Neither myocardial infarction nor acute kidney injury occurred in the study population.

## Discussion

In this prospective observational trial, changes in COP between a gelofusine, albumin or crystalloid based prime fluid strategies, the last two either combined with RAP, were measured. The decrease in COP was the most distinct in the gelofusine group, compared with albumin or crystalloid. Importantly, COP was inversely correlated with fluid balance, -requirements and duration of mechanical ventilation. Haemodilution, defined by haematocrit, was better preserved in the crystalloid group compared with gelofusine and albumin groups. These findings suggest that, especially in the early phase of CPB, interstitial fluid accumulation is associated with haemodilution which lowers COP.

Haemodilution reduces the oxygen carrying capacity due to lower haematocrit, blood viscosity^14^ and capillary diffusion capacity, which are all important for organ perfusion and function.^15^ Moreover, haemodilution lowers COP and causes interstitial fluid accumulation which could compromise tissue oxygenation and organ perfusion further.^2^ The key factor here seems to be the microcirculation.^16^ Several attempts have been made to improve capillary perfusion, e.g., by removal of fluids through diuretics^17^ or increasing blood viscosity,^14^ suggesting that prevention of fluid accumulation during CPB with high-viscosity colloids could preserve capillary perfusion. COP plays a key role in fluid accumulation and is seen as reliable indicator of haemodilution in cardiac surgery.^1^ Yet, the threshold at which edema occurs is controversial. A previous study showed that COP <15 mmHg during CPB was correlated with fluid overload.^18^ With a lowest mean COP of 16.4 mmHg in our study, the COP threshold for fluid overload seems to be higher since the inverse correlation with fluid balance, -requirements and duration of mechanical ventilation exists. In contrast with previous studies,^18–20^ COP did not correlate with blood loss, weight gain or hospital stay. However, our study was not powered for these parameters, and unfortunately, weighing patients was not possible in our study.

The observed differences in COP between groups may have been influenced by several factors due to protocol differences between institutions. First, prime volume was not equal between groups due to differences in CPB tubing. Second, patients in the gelofusine group received cold crystalloid cardioplegia and patients in other groups received warm blood cardioplegia. This resulted in more haemodilution after aortic cross clamping in the gelofusine group. Consequently, COP may have decreased further after cardioplegia administration, resulting in increased interstitial fluid accumulation. Third, the use of RAP in prime fluids with crystalloids or albumin could have preserved COP during CPB, since lower prime volumes were used initially as result of RAP. This may have resulted in less haemodilution and subsequently higher COP compared with gelofusine.

CPB induced haemodilution resulted in a decrease of haematocrit, especially with gelofusine and albumin. The use of RAP does not fully explain the difference in haematocrit after CPB initiation between the crystalloid and colloid groups, since RAP was also applied (*n* = 14) in the albumin group, not in the gelofusine group, and crystalloid cardioplegia was only administered in the gelofusine. The use of colloids combined with RAP could have led to a more profound haemodilution in the albumin group. Transfusion rates were comparable, however. The higher amount of cell saver blood in the gelofusine group, could have led to the increased haematocrit after CPB. RAP has been found to lower fluid balance and decrease transfusion rates in previous studies,^8,9,21^ which is confirmed by the guidelines on patient blood management for adult cardiac surgery.^10^ Importantly, this study was not powered to find differences in transfusion rates and differences in fluid balance could be the result of several factors in this study, i.e. COP, prime volume, cardioplegia, strategy for fluid resuscitation, etcetera.

This study has several limitations. It was conducted to explore the role of COP between different prime fluid strategies. It was not powered to detect differences in patient outcome, nor to provide a recommendation for an optimal prime fluid strategy. The multicenter aspect of this study is rather a flaw then an advantage due to differences in institutions protocols. Consequently, prime volume, cardioplegia type and volume differed between groups. Also, the addition of RAP in the albumin group hampers the interpretation of the present results. A large, single center and randomized study would have eliminated these biases and would have been needed to answer these questions. Protocol violation occurred in 1 patient. RAP was mistakenly performed in one patient in the gelofusine group.

In order to decrease the burden on intensive care capacity during the COVID pandemic, a ‘fast-track protocol’ was implemented in the LUMC, wherein patients were extubated in the operation room in order to continue cardiac surgery care, without intensive care capacity. This resulted in shorter duration of mechanical ventilation. Subsequently, the correlation between COP and duration of mechanical ventilation in this study could be debated. Additionally, in the Amsterdam UMC location Vrije Universiteit Amsterdam more patients with other relevant comorbidities were operated, compared with the LUMC, although EuroSCORE and ASA score were similar.

In summary, our data suggest that particularly in the early phase of CPB, haemodilution lowers COP leading to interstitial fluid accumulation. The decrease in COP was the most distinct in the gelofusine group. However, the effect of crystalloid cardioplegia and absence of the use of RAP in the gelofusine group on COP could not be measured separately herein. Haemodilution is inevitable in on-pump cardiac surgery but information on an optimal prime fluid strategy to preserve microcirculatory perfusion is lacking. The results of this study indicate that COP could be a valuable parameter in future (randomized) studies with different fluids but comparable priming techniques and volumes, eliminating the shortcomings of the present study.

## Supplemental Material

Supplemental Material - Changes in colloid oncotic pressure during cardiac surgery with different prime fluid strategies Supplemental Material for A conceptual framework for characterising lifecourse determinants of multiple long-term condition multimorbidity by Anne Maria Beukers, Juan de Villiers Hugo, Renard Gerardus Haumann, Jan Willem Taco Boltje, Evy Loan Khiam Ie, Stephan Alexander Loer, Carolien Suzanna Enna Bulte and Alexander Vonk in Perfusion
