# Supplementary material for: Changes in colloid oncotic pressure during cardiac surgery with different prime fluid strategies
Source: Perfusion. 2023 Aug 8;39(7):1371–9. doi: 10.1177/02676591231193626 (PMC11448106; doi:10.1177/02676591231193626)
Supplement: Supplemental Material - Changes in colloid oncotic pressure during cardiac surgery with different prime fluid strategies [file sj-pdf-1-prf-10.1177_02676591231193626.pdf]

Supplementary online content

Appendix 1. Anesthesia protocol

Appendix 2. Cardiopulmonary bypass protocol

Appendix 3. COP

Table 1. Continued antiplatelet therapy

Appendix 1. Anesthesia protocol

Amsterdam UMC location Vrije Universiteit Amsterdam

On the day before surgery, patients received sleeping medication (temazepam 10 or 20 mg, depending on their weight ≤60 kilogram (kg) or >60 kg respectively) and promethazine 25 mg. Following a morning dose of oxazepam 10 or 20 mg, depending on their weight as described above, anesthesia was induced using intravenous sufentanil (1-3 $\mu g.kg^{-1}$), midazolam (0.1 $mg.kg^{-1}$), combined with rocuronium (0.5-1.0 $mg.kg^{-1}$) and maintained by continuous propofol infusion (6-8 $mg.kg^{-1}$ per hour). Patients received dexamethasone (1 $mg.kg^{-1}$), tranexamic acid (1g) and cefazolin (2g).

LUMC

On the day before surgery, patients received sleeping medication (lorazepam 1 mg sublingual (s.l.) or 2 mg s.l., depending on their age ≤65 years or > 65 years respectively). On the day of surgery patients were not allowed to take their anti-diabetics, diuretics, ACE inhibitors and AT-II blockers. One hour before surgery patients were premedicated with lorazepam 1 mg s.l. or 2 mg s.l., depending on their age (≤65 years or > 65 years, respectively). Patients on long-term steroid therapy were administered perioperative stress doses of corticosteroids.

Anesthesia was induced by using combinations (depending on the anesthesiologist) of intravenous opioids (sufentanil and/or remifentanil), hypnotics (propofol, ketamine or midazolam) together with rocuronium bromide and maintained by continuous infusion of propofol and sufentanil and, depending on the anesthesiologist, remifentanil. In the majority of cases target controlled infusion (TCI) was used. Dosage of anesthetics were adjusted as necessary to achieve a bispectral index (BIS) between 40 and 60. After induction of anesthesia tranexamic acid (1g) and cefazolin (2g) were given.

Appendix 2. Cardiopulmonary bypass protocol

Amsterdam UMC location Vrije Universiteit Amsterdam

A C5 or S5 heart-lung machine (LivaNova Nederland NV, Amsterdam, the Netherlands) with a centrifugal pump and a heater-cooler device (LivaNova Nederland NV, Amsterdam, the Netherlands) was used for CPB. The bypass circuit consisted of a heparin-coated polyvinyl tubing system with a hollow-fiber oxygenator and arterial line (Affinity, Medtronic, Minneapolis, MN/LivaNova Nederland NV, Amsterdam, the Netherlands), a soft shell venous reservoir (MVR 1600, Medtronic, Minneapolis MN/LivaNova Nederland NV, Amsterdam, the Netherlands) and a BB Fusion CVR cardiotomy reservoir (Medtronic, Minneapolis MN/LivaNova Nederland NV, Amsterdam, the Netherlands). The bypass circuit was primed according based on the study groups. CPB was initiated after administration of heparin (300-500 IE.kg^{-1}) and when the activated clotting time exceeded 420 s (Hemochron Signature Elite, Edison, USA). Blood flow during mild hypothermic (34-36° Celsius) CPB will be kept between 2.2-2.6 L.min^{-1}.m$_2$$^{-1}$. Myocardial protection was achieved using cold crystalloid cardioplegia solution (St. Thomas, 4° Celsius) and weaning from CPB was started when the rectal temperature had reached 36° Celsius. A cell saving device (Autolog, Medtronic, MN, USA) was used for re-transfusion of pericardial shed blood. After weaning from CPB, protamine in a 0.7:1 fashion to reverse heparin and tranexamic acid (2g) were given.

In case of vasoplegia, defined by when hematocrit falls below 0.24 L.L^{-1} during CPB, patients received in order 250 mL gelofusine, 800 mL LR, 20 g human albumin, 800 mL LR and so on based on the decision of the perfusionist. Before and after CPB patients received LR based on the decision of the anesthesiologist.

LUMC

In the albumin + LRs and LRs + RAP groups, an S5 heart-lung machine with a C5 centrifugal drive unit (LivaNova, Mirandola, Italy) was used for CPB. The bypass circuit consisted of a custom made set for LUMC and includes a heparin-coated polyvinyl tubing system with a

hollow-fiber oxygenator Quadrox-i-Adult (Maquet Cardiopulmonary, Rastatt, Germany) or a Terumo Capiox FX15 (Terumo Corporation, Tokyo, Japan), a soft shell venous reservoir and a cardiotomy reservoir from respective companies. Temperature management was achieved by regulating water taps from hospital sanitation. CPB was initiated after administration of heparin (3-5 $mg.kg^{-1}$) and when the activated clotting time (ACT) exceeded 400 s. Blood flow during mild hypothermia (32-36°C) was be kept between 2.0-2.4 l/min/m2 to provide adequate organ perfusion. Myocardial protection was achieved by intermitted antegrade warm (patient temperature) blood cardioplegia solution with KCL 15% (Calafoire). Weaning from CPB started when the nasopharyngeal temperature had reached 36,5°C. A cell saving device (Xtra, LivaNova, Mirandola, Italy) was used for transfusion of pericardial shed blood. After weaning from CPB, administration of protamine in a 0.8-:1 fashion to reverse heparin and 2 g of tranexamic acid were given.

Retrograde autologous priming LUMC

There was not a strict protocol on the amount of volume that was displaced, but rather a multifactorial approach with the hemodynamic stability of the patient at its center whereas it was tried to utilize RAP in entire cardiac surgery population.

Both groups applied RAP using clinical parameters such as central venous pressure , mean arterial pressure and intracardiac filling pressure based on transesophageal echocardiography as guidance to the amount of fluid displaced. Additional phenylephrine was administered to preserve mean arterial pressure during RAP. Once the desired amount of prime was displaced the transfusion bag was clamped and CPB was started. If additional fluids were needed during CPB to maintain optimal organ perfusion, displaced prime was used. In case of vasoplegia, defined by when hematocrit falls below 0.24 $L.L^{-1}$ during CPB, patients received crystalloids or colloids based on the decision of the perfusionist.

Appendix 3: COP

COP was determined from plasma with the Osmomat 050. The plasma samples were injected with a syringe through a rubber septum into the cell of the Osmomat 050. The measuring cell was automatically rinsed with LR solution and was separated in two halves by a semipermeable membrane through which only water and electrolyte molecules can permeate. Due to the oncotic pressure difference, solvents from the measuring cell entered into the upper cell until an equilibrium was reached. An electronic pressure measuring system in the measuring cell transduced the negative pressure into an electric signal that was shown on the display in mmHg.

Table 1. Additional patient characteristics

	Gelofusine (n=20)	Crystalloids (n=20)	Albumin (n=20)	*p*-value
**Continued antiplatelet therapy**				
Acetylsalicylic acid	16 (80%)	12 (60%)	13 (65%)	0.367
Clopidogrel	2 (10%)	5 (25%)	7 (35%)	0.170
Ticagrelor	0	0	0	-
Not applicable	4 (20%)	7 (35%)	7 (35%)	0.490
Other	0	1 (5%)	0	0.362
**Discontinued antiplatelet therapy**				
Acetylsalicylic acid	0	0	0	-
Clopidogrel	2 (10%)	2 (10%)	0	0.343
Ticagrelor	1 (5%)	1 (5%)	0	0.596
Not applicable	16 (80%)	16 (80%)	17 (85%)	0.895
Other	1 (5%)	1 (5%)	3 (15%)	0.428

Means compared with One-way ANOVA. †Medians compared with non-parametric independent-samples median test. Frequencies compared with Chi-square test *statistically significant value $P < 0.05$
